# What is the role of paternal genetic transmission on risk for PTSD and internalizing and externalizing disorders?

**DOI:** 10.1017/S0033291725000546

**Published:** 2025-03-10

**Authors:** Ananda B. Amstadter, Linda Abrahamsson, James E. Hart, Jan Sundquist, Kristina Sundquist, Kenneth S. Kendler

**Affiliations:** 1 Virginia Institute for Psychiatric and Behavioral Genetics, Virginia Commonwealth University, Richmond, VA, USA; 2 Department of Psychiatry, Virginia Commonwealth University, Richmond VA, USA; 3 Center for Primary Health Care Research, Department of Clinical Sciences, Lund University, Malmö, Sweden; 4 University Clinic Primary Care Skåne, Region Skåne, Sweden

**Keywords:** posttraumatic stress disorder, substance use disorder, major depressive disorder, genetic epidemiology

## Abstract

**Background:**

We utilize a novel contrastive genetic-epidemiological method, the Maternal Half-Sibling Families with Discordant Fathers (MHSFDF) design, to examine cross-generational genetic transmission of posttraumatic stress disorder (PTSD) and related internalizing major depression (MD), and externalizing disorders: alcohol use disorder (AUD) and drug use disorder (DUD).

**Methods:**

Using Swedish national registries, we identified 72,467 maternal half-sibling pairs reared together whose biological fathers were discordant for the diagnoses of PTSD, MD, AUD, and DUD. Offspring selected had to have less than 1 year of contact with their affected fathers. We examined the differences in outcome for within- and cross-disorder risk of diagnosis in the half-siblings with an affected versus unaffected father.

**Results:**

Paternal PTSD increased the risk of PTSD (HR: 1.43, 95% CI: 1.05–1.96) and MD (HR: 1.55, CI: 1.28–1.88) in offspring. It did not, however, elevate the risk of externalizing disorders (AUD or DUD). Offspring of fathers with AUD, DUD, or MD had increased risk of PTSD, suggesting sharing of vertically transmitted genetic risk between these disorders. No sex effects were found for any studied diagnosis.

**Conclusions:**

This study is the first to show cross-generation genetic transmission for PTSD using the MHSFDF design. The pattern of cross-disorder genetic risk broadly supported an internalizing versus externalizing disorder split.

Posttraumatic stress disorder (PTSD) is as a prevalent and often severe psychiatric condition, with a lifetime prevalence of 5.4–6.0% of the United States population; notably, lifetime prevalence is markedly higher among females (7.9–9.1%) compared to males (2.5–3.1%) (Kessler et al., 2012). PTSD is highly burdensome, as symptoms of PTSD, such as intrusive thoughts, hypervigilance, and avoidance of trauma reminders, and physical correlates, like chronic pain, are associated with substantial impairments in social and occupational functioning (Kessler et al., 2005; Ryder, Azcarate, & Cohen, 2018; Taft et al., 2011). Notably, PTSD is highly comorbid with internalizing disorders, such as major depressive disorder (MD) (Rytwinski, Scur, Feeny, & Youngstrom, 2013), and externalizing disorders like drug use disorder (DUD) and alcohol use disorder (AUD). Given the impact of PTSD and related comorbidities, efforts to understand its etiology are needed.

PTSD has been shown from multiple designs to be influenced by genetic risk *within the same generation.* Twin studies, aiming to clarify whether the familial association is the result of genetic or environmental effects, have estimated the lifetime genetic liability for developing PTSD following trauma exposure to be 26% (95% CI = 12–40) in an all-male veteran sample (Koenen et al., 2008), and 38% (CI: 24–52%) in a mixed-sex civilian cohort (Stein et al., 2002). On the higher range of estimates is a heritability of 72% (95% CI 41–85) in an all-female cohort (Sartor et al., 2011). Our recent twin study, utilizing the Swedish medical registries, formally tested for sex differences in the genetic heritability of PTSD, confirming that the heritability is greater in females (35% CI: 33–38%) than in males (28.6%, CI: 26–32%) and further found evidence of a qualitative sex effect such that the genetic correlation between the sexes was significantly different than unity (rg = .81, CI: .73–.89) (Amstadter et al., 2024). Molecular genetic studies have also demonstrated evidence of heritability of PTSD (h^2^_SNP_ = .053, s.e. = .002) (Nievergelt et al., 2024).

PTSD has also been shown to be transmitted in families *across generations.* As summarized in a systematic review, the majority of family studies demonstrated a significant association between parent and offspring posttraumatic stress symptoms following medically and non-medically related traumatic events (Leen-Feldner et al., 2013). Family studies are constrained by the challenge of untangling genetic and environmental influences due to the shared environment of intact families. Adoption models observe individuals who are raised in environments separate from their biological parents, enabling a clearer separation of genetic and rearing effects. To our knowledge, only one such adoption study has been conducted examining cross-generational transmission of PTSD; our recent extended-adoption study (which expands families to include stepparents who raise non-biological children) in the Swedish medical registries observed the extent and origins of intergenerational transmission of adverse stress reactions and PTSD. Notably, approximately half of the similarity between parent and child for adverse stress responses and PTSD is attributed to rearing factors, while the remainder is due to genetic factors (Amstadter et al., 2024).

Observational designs, such as adoption studies, provide valuable insight into intergenerational transmission of disorders, but they are not without biases due to screening of adoptive parents, resulting in non-representative samples (e.g. higher socioeconomic status, lower mental health problems) (Cadoret, 1986). Adoption designs are also limited by potential assertive placement, intra-uterine effects, and early contact between biological parents prior to placement of adoptees in foster homes and/or with adoptive parents. To provide robust findings in observational sciences, it is necessary to use a variety of methods with separate sets of potential biases to answer similar underlying questions, referred to as triangulation (Munafò & Davey Smith, 2018). One such alternative perspective in genetic epidemiology is that of the Maternal Half-Sibling Families with Discordant Fathers (MHSFDF) design, recently used by our group (Kendler, Ohlsson, Sundquist, & Sundquist, 2020). This contrastive method identifies pairs of maternal half-siblings, reared together, whose fathers are discordant for the phenotype of interest and the one relevant half-sibling has not lived with the affected father. Thus, maternal genetic influence and rearing environment is shared between the offspring, with the siblings differing only in paternal genetic influences. Therefore, like a classical adoptive study, this design can be used to isolate and thereby clarify the influence of paternal genetic risk on the development of medical and psychiatric conditions. Given that families in this design are not screened, the design is not affected by the same limitations as adoption studies, and thus, offers a different vantage point to examine cross-generational transmission. Prior work in the Swedish Registries has demonstrated that paternal genetic risk increases the risk of offspring MD, AUD, and DUD, and further, that substantial sharing of genetic risk exists between AUD and DUD, and less so for MD (Kendler, Ohlsson, Sundquist, & Sundquist, 2020). This work has yet to be extended to PTSD.

We utilized the MHSFDF design to (a) clarify the nature of paternal genetic risk for PTSD and (b) examine the nature of the cross-generational cross-disorder paternal genetic transmission. Given that PTSD is highly comorbid with internalizing disorders, such as MD, and externalizing disorders, such as AUD and DUD, and shares genetic risk with these conditions, this is an important extension of the current literature.

## Methods

Information for this study was collected from nationwide Swedish registers (see Supplement). Ethical approval was obtained from the authorities. Each person’s unique identification number, having been replaced with serial numbers for confidentiality, was used for registry linkages. We made use of the Multigenerational register to search for all Swedish-born maternal half-sibling pairs having birth years 1960–1990, no more than 7 years in age difference, and both still residing in Sweden at age 16. Furthermore, the half-siblings had to reside with their biological mother between ages 0–15 (information taken from the Population and Housing Census and the Total Population Register). In total we found 72,467 such pairs, consisting of 126,522 unique individuals. For the half-sibling pairs, and their two different biological fathers, we searched for diagnoses of PTSD, MD, AUD, and DUD using the Swedish Hospital Discharge Register, Outpatient Care Register, almost nationwide primary care data, the Swedish Prescribed Drug Register, the Swedish Cause of Death Register, the Swedish Criminal Register, and the Swedish Suspicion Register. For details on how diagnoses were defined, see Supplementary Table 1.

Four different sets of half-sibling pairs were then defined, being groups with discordant biological fathers for PTSD, MD, AUD, or DUD, one group for each paternal disorder, consisting of 1,752, 8,888, 15,295, and 5,224 maternal half-sibling pairs, respectively. The offspring lived for no longer than 1 year with an affected father, to restrict environmental influence of affected fathers. Notably, inverse analyses examining maternal genetic risk were not possible, as there were not sufficient numbers of paternal half-sibling pairs with discordant biologic mothers.

In multivariable stratified Cox proportional hazards regression models, we studied lifetime diagnoses of PTSD, MD, AUD, or DUD in biological father, on time to first diagnosis (of PTSD, MD, AUD, or DUD) in offspring. Hazard ratios (HRs) refer to the potential increase in risk in the child with the affected father compared to their half-sibling with an unaffected father. We made use of separate stratums for each half-sibling pair to adjust for unmeasured shared factors among the half-siblings. Offspring age was used as time scale and individuals were followed from age five, or from 1987-01-01, whichever occurred lastly, to first diagnosis, emigration, death, or study end of 2018-12-31, whichever occurred firstly. The year 1987 was chosen for reasons of availability of PTSD data in registries. Models were controlled for sex and birth year of child and birth year in father. To further evaluate any sex-specific effects, all models were run with additional interaction effects between the exposure of having an affected biological father, with the sex of the child. The 16 interaction effects were evaluated at Bonferroni-corrected levels of 0.05/16 = 0.003. To evaluate differences in effects dependent on type of paternal diagnosis, all data were included in the same model with separate effects for the different paternal diagnoses and with robust variance to take into account offspring included more than once. We calculated contrasts of differences between paternal diagnoses for testing whether they differed at Bonferroni-corrected levels of 0.05/24 = 0.002.

Data analysis was conducted from February 27, 2024, to October 31, 2024. Statistical analyses were performed using R, version 4.4.0 (R, 2024) (Supplementary Table 2) and SAS, version 9.4 (Inc., 2016).

## Results

The number of informative pairs ranged from 1,752 for PTSD to 15,295 for AUD; see Table 1. The average ages of affected versus unaffected fathers were very similar. As expected, the prevalence of all disorders in the offspring was higher among the affected father compared to the unaffected father across all disorder types. Furthermore, the prevalence of all disorders was higher among these offspring compared to the Swedish general population.

**Table 1.** Sample descriptives of general population and parent–offspring pairs

	General population	Offspring within half-sibling pairs	PTSD		MD		AUD		DUD	
			Unaffected father	Affected father	Unaffected father	Affected father	Unaffected father	Affected father	Unaffected father	Affected father
Number of offspring	3,124,814	126,522	1,752	1,752	8,888	8,888	15,295	15,295	5,224	5,224
Mean YoB father (sd)	1944.4 (10.6)	1947.6 (9.0)	1952.1 (8.7)	1952.7 (8.1)	1949.0 (9.0)	1948.6 (8.8)	1948.2 (9.0)	1947.6 (8.6)	1951.3 (8.5)	1951.5 (7.9)
Mean YoB mother (sd)	1947.4 (10.0)	1950.6 (7.8)	1954.8 (7.3)	1954.8 (7.3)	1951.7 (7.9)	1951.7 (7.9)	1950.9 (7.9)	1950.9 (7.9)	1954.1 (7.3)	1954.1 (7.3)
Mean YoB child (sd)	1974.8 (9.1)	1975.5 (8.0)	1980.6 (7.4)	1978.0 (7.4)	1977.7 (7.9)	1974.9 (8.0)	1976.8 (7.9)	1973.8 (7.9)	1979.6 (7.4)	1976.9 (7.5)
% Males	51.3	50.7	50.2	49.3	51.0	50.7	51.4	51.0	51.0	51.5
PTSD in child (%)	6.5	9.0	10.2	13.7	9.3	11.9	9.1	11.5	9.8	13.0
MD in child (%)	17.2	22.6	23.6	32.6	23.2	30.4	23.5	28.2	25.0	31.3
AUD in child (%)	4.8	8.0	8.1	10.8	8.1	10.3	7.9	13.2	8.7	14.2
DUD in child (%)	5.0	9.0	13.6	14.3	10.4	12.1	9.7	13.4	13.7	19.7
Average parental level of education^a^										
1. Pre-secondary education, No. (%)	464,521 (14.9)	16,947 (13.4)	151 (8.6)	123 (7.0)	1,135 (12.8)	982 (11.0)	2,289 (15.0)	2,032 (13.3)	614 (11.8)	451 (8.6)
2. Secondary education, No. (%)	1,575,482 (50.4)	76,035 (60.1)	1,120 (63.9)	1,063 (60.7)	5,484 (61.7)	5,394 (60.7)	9,514 (62.2)	9,367 (61.2)	3,274 (62.7)	2,953 (56.5)
3. Post-secondary education, No. (%)	1,083,608 (34.7)	33,537 (26.5)	481 (27.5)	566 (32.3)	2,268 (25.5)	2,512 (28.3)	3,492 (22.8)	3,896 (25.5)	1,336 (25.6)	1,820 (34.8)

*Note:* year of birth (YoB), standard deviation (sd), posttraumatic stress disorder (PTSD), major depressive disorder (MD), alcohol use disorder (AUD), drug use disorder (DUD).

a When the average level fell between two categories, a rounding off to the higher educational level was performed.

**Figure 1.**
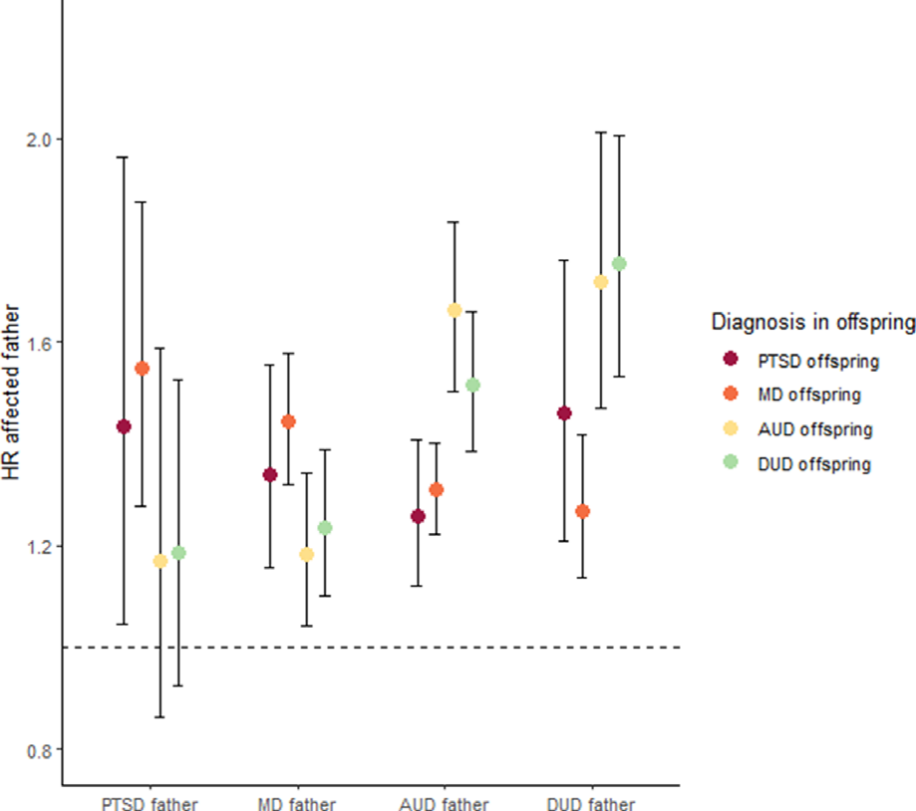
Prediction of risk in offspring from maternal half-sibling families with fathers discordant for PTSD, MD, AUD, and DUD. *Note:* Hazard ratios and 95% CIs are presented, posttraumatic stress disorder (PTSD), major depressive disorder (MD), alcohol use disorder (AUD), drug use disorder (DUD).

The results from the stratified Cox proportional hazard models, controlling for birth years of offspring and father, and sex of the child, along with 95% CIs, are shown in Table 2 and Figure 1. PTSD in fathers increased the risk for PTSD (1.43, 95% CI: 1.05–1.96) and MD (1.55, 95% CI: 1.28–1.88) in offspring. PTSD in fathers did not increase risk for the externalizing disorders (i.e. AUD or DUD) in offspring. Cross-disorder transmission on risk for PTSD was also estimated. The risk of PTSD in offspring was higher among offspring whose fathers had AUD (1.26, 95% CI: 1.12–1.41), DUD (1.46, 95% CI: 1.21–1.76), and MD (1.34, 95% CI: 1.16–1.56), suggesting sharing of vertically transmitted genetic risk. As can be seen in Figure 1, the pattern of cross-disorder genetic transmission is broadly consistent with an internalizing versus externalizing disorder split. However, for risk of PTSD in offspring, we could not detect at Bonferroni-corrected levels (*p* < 0.002), any significant differences depending on type of paternal diagnosis. Risk for MD in offspring is better predicted by PTSD than it is the externalizing disorders (i.e. AUD, DUD) and is better predicted by MD than it is AUD. The overall pattern was the opposite for the externalizing disorders; risks of AUD and DUD are better predicted by externalizing disorders than internalizing disorders, the exception being risk of DUD in offspring, where we could not detect a significantly higher effect of AUD in comparison to PTSD. From these results, we can broadly conclude that PTSD is more genetically aligned with MD than it is AUD and DUD.

**Table 2.** Stratified Cox regression models, controlled for birth years of offspring and father and sex of child

Outcome	PTSD in biological father	MD in biological father	AUD in biological father	DUD in biological father
PTSD in child	1.43 (1.05–1.96)	1.34 (1.16–1.56)	1.26 (1.12–1.41)	1.46 (1.21–1.76)
MD in child	1.55 (1.28–1.88)	1.44 (1.32–1.58)	1.31 (1.22–1.40)	1.27 (1.14–1.42)
AUD in child	1.17 (0.86–1.59)	1.18 (1.04–1.34)	1.66 (1.50–1.84)	1.72 (1.47–2.01)
DUD in child	1.19 (0.92–1.53)	1.24 (1.10–1.39)	1.52 (1.38–1.66)	1.75 (1.53–2.01)

*Note:* posttraumatic stress disorder (PTSD), major depressive disorder (MD), alcohol use disorder (AUD), drug use disorder (DUD)

As shown in Table 3, at Bonferroni-corrected levels, we did not find any significant sex-specific effects of having a biological father being affected by any of the four diagnoses of interest.

**Table 3.** Stratified Cox regression models, controlled for birth years of offspring and father and sex of child, including interaction effects between having an affected father and sex of offspring

Outcome in offspring	Sex of offspring	PTSD in biological father	MD in biological father	AUD in biological father	DUD in biological father
PTSD	Male	1.57 (0.91–2.71)	1.13 (0.88–1.46)	1.35 (1.11–1.64)	1.86 (1.33–2.58)
	Female	1.35 (0.87–2.09)	1.52 (1.23–1.88)	1.19 (1.01–1.40)	1.23 (0.94–1.61)
*p*-value		0.69	0.11	0.37	0.08
MD	Male	1.74 (1.26–2.41)	1.53 (1.32–1.76)	1.35 (1.21–1.51)	1.46 (1.22–1.74)
	Female	1.42 (1.08–1.86)	1.38 (1.21–1.57)	1.28 (1.16–1.41)	1.13 (0.96–1.32)
*p*-value		0.37	0.33	0.49	0.048
AUD	Male	1.22 (0.78–1.89)	1.30 (1.08–1.56)	1.94 (1.68–2.24)	1.70 (1.36–2.13)
	Female	1.09 (0.52–2.30)	1.02 (0.80–1.30)	1.33 (1.11–1.59)	1.75 (1.30–2.34)
*p*-value		0.81	0.15	0.0032	0.91
DUD	Male	1.08 (0.75–1.55)	1.20 (1.01–1.42)	1.57 (1.38–1.79)	1.92 (1.58–2.33)
	Female	1.35 (0.87–2.09)	1.29 (1.04–1.60)	1.43 (1.22–1.69)	1.53 (1.20–1.95)
*p*-value		0.48	0.62	0.42	0.19

*Note:* posttraumatic stress disorder (PTSD), major depressive disorder (MD), alcohol use disorder (AUD), drug use disorder (DUD)

## Discussion

To our knowledge, this is the first application of this novel MHSFDF design on risk for PTSD in offspring. This novel genetic epidemiologic design allows for the estimation of the influence of paternal genetic risk on offspring outcomes, and it has been applied in a prior study to understand cross-generational transmission of MD, AUD, and DUD (Kendler, Ohlsson, Sundquist, & Sundquist, 2020). This study consists of similar samples of half-sibling pairs, with the difference that follow-up data have now been extended by several years and has more complete coverage of primary care data, thereby increasing prevalence of especially MD but also DUD. Similar differences in MD prevalences can be seen in the literature from studies using the registries used herein, where later papers were able to use prolonged follow-up periods, for example, comparing (Kendler et al., 2018) to Kendler (Kendler et al., 2022b). This is a contrastive design, comparing risk between maternal half-siblings who are raised together without the affected father. These offspring share maternal genetic risk and share a similar rearing environment and experience of divorce, which itself is associated with intergenerational transmission of some disorders such as AUD (Salvatore, Aggen, & Kendler, 2022), enabling a specific examination of the magnitude of paternal genetic transmission. Using the Swedish national registries, we identified pairs of half-siblings whose fathers were discordant for PTSD to examine if paternal genetic risk for PTSD was transmitted to offspring and its magnitude. We were also interested in cross-disorder transmission of risk, and thus, we identified pairs whose fathers were discordant for classic internalizing (i.e. MD) and externalizing (i.e. DUD, AUD) disorders. Notably, inverse analyses examining maternal risk were not possible due to low numbers of pairs. Our study yielded three main findings to be discussed in turn.

First, we found that PTSD showed cross-generational transmission, which replicated prior evidence for cross-generational genetic transmission of PTSD using an adoption design (e.g. (Amstadter et al., 2024). Notably, the HR in our adoption analyses, when adjusted for birth year and sex, reflecting genetic transmission that included both maternal and paternal genetic risk was very similar (HR = 1.35, 95% CI 1.23–1.48) to what we found in these analyses, which suggests that our (novel) findings are robust. A prior MHSFDF study of AUD and DUD also found overlapping CIs between the half-sibling design and the extended adoption design (Kendler et al., 2022a; Kendler, Ohlsson, Sundquist, & Sundquist, 2020). Of note, our study is the first to isolate the paternal genetic effects on PTSD transmission. Our finding is also consistent with twin studies demonstrating moderate heritability for PTSD (Sartor et al., 2011; Stein et al., 2002; True et al., 1993; Wolf, Mitchell, Koenen, & Miller, 2014). Notably, the HR for risk for PTSD in offspring with an affected father was similar to that found in a prior MHSFDF study of MD (Kendler, Ohlsson, Sundquist, & Sundquist, 2020). As highlighted above, genetic epidemiology is an observational science, and thus, consistent results across methods with different assumptions/biases is important for the progression of evidence.

Our second main finding stems from the cross-generational cross-disorder analyses. These analyses demonstrated an interesting pattern broadly indicative of an internalizing versus externalizing division, which can be seen in Figure 1. However, at Bonferroni-corrected levels, the risk of PTSD in offspring was not differentially predicted by type of paternal diagnosis. Risk for MD in offspring was better predicted by PTSD than it was the externalizing disorders. Results for the externalizing disorders showed the opposite pattern, wherein the risk conferred by paternal risk for AUD and DUD increase risk for offspring AUD and DUD to a greater degree than it does for PTSD and MD; with the exception being risk of DUD in offspring was not significantly different between AUD and PTSD, consistent with prior analyses from the registry (Kendler, Ohlsson, Sundquist, & Sundquist, 2020) and is expected given prior studies suggesting genetic overlap between AUD and DUD (Kendler et al., 2011; 2022a). Although both twin and molecular studies show that PTSD is genetically correlated with AUD and DUD (Sheerin et al., 2020; Xian et al., 2000), the magnitude of the genetic correlation is substantially lower than that of PTSD and MD, which is near unity (Nievergelt et al., 2024; Sartor et al., 2012). Our findings are also consistent with twin studies on the latent categorization of psychiatric disorders via internalizing and externalizing dimensions, showing that although PTSD genetically loads onto both internalizing and externalizing dimensions, the loading is nearly twice as strong on the internalizing than the externalizing dimension (Wolf et al., 2010). Additionally, molecular genetic data examining genetic correlations between PTSD and internalizing versus externalizing disorders also find a higher genetic correlation between PTSD and internalizing disorders in comparison to externalizing disorders (Nievergelt et al., 2024). Furthermore, genomic structural equation modeling approaches have found a moderate genetic correlation between PTSD and AUD (Bountress et al., 2022). Another gSEM analysis found that PTSD genetically loaded onto two correlated factors, an ‘internalizing’ factor and a ‘neurodevelopmental disorders’ factor (Grotzinger et al., 2022). Notably, these approaches have yet to model drug use disorders.

Third, we found no differential impact of paternal genetic risk for any of the disorders on offspring risk by sex; however, the lack of effect could be due to lower power to study sex-specific effects. Thus, our findings are not indicative of a cross-generational qualitative sex effect. The only twin study, to date, on PTSD to test for sex effects was conducted by our group in the registry data (Amstadter et al., 2024), finding evidence of both a quantitative (i.e., higher heritability in females versus males) and qualitative sex effect. The genetic correlation between females and males was quite high (rg = .81, 95% CI: .73–.89) but could not be constrained to unity, suggesting a modest qualitative sex effect for PTSD. In light of sex differences both in the genetic etiology of PTSD and in the epidemiology of PTSD (Kilpatrick et al., 2013; Tolin & Foa, 2008), further investigation of sex differences is warranted.

Our findings should be interpreted in light of a number of limitations. PTSD and MD cases were primarily detected from primary care data, which likely does not capture all of the cases. AUD and DUD diagnoses are based on seeking medical attention or on being involved with the criminal justice system, and thus, also likely represent an underestimation. Many prior analyses of these definitions have been conducted and support the validity of the definitions with evidence of high concordance across methods (Kendler, Ohlsson, Sundquist, & Sundquist, 2015) and behavioral genetic results of familial associations replicating those of generated from clinical interview studies (Prescott & Kendler, 1999; Tsuang et al., 1996). Our findings may generalize more for severe substance use disorder cases given the threshold for being classified with DUD or AUD via registration is high and thus, more mild or moderate cases may not have been detected. Additionally, this design is not without assumptions (e.g., similar rearing environment for the half-siblings) that could have influenced our findings. Limitations to generalization outside of the Swedish population and outside of divorced families also exist. We note that the prevalence of all disorders studied was higher among the offspring compared to the general population; this is not entirely surprising, as prior work has demonstrated a role of divorce in intergenerational transmission of AUD (Salvatore, Aggen, & Kendler, 2022). Despite these limitations, our study has methodological advantages (e.g., no positive selection bias of screened parents for health as in adoption studies, lack of influence of self-report bias or recall bias), and offers a unique ability to examine cross-generational genetic transmission.

In conclusion, our results showed that PTSD has a cross-generational transmission from paternal genetic risk. We also found evidence of cross-generational cross-disorder transmission, broadly consistent with an internalizing versus externalizing pattern.

## Supporting information

Amstadter et al. supplementary material Amstadter et al. supplementary material

## Data Availability

The data are not publicly available due to legal restrictions with regard to the nationwide Swedish registers but could be required directly from the responsible authorities after their approval.

## Supplementary material

The supplementary material for this article can be found at http://doi.org/10.1017/S0033291725000546.

## Originality statement

All information and material in the manuscript are original.
